# The gatekeeper: how PSY1 controls root growth

**DOI:** 10.1093/plcell/koag194

**Published:** 2026-06-29

**Authors:** Andrea Gómez-Felipe

**Affiliations:** Assistant Features Editor, The Plant Cell, American Society of Plant Biologists, United States; Department of Biology, Indiana University, Bloomington, IN 47405, United States

The growth of plant organs is a masterclass in spatial coordination, requiring a precise balance between cell division, elongation, and specialization. In the primary root of the model plant *Arabidopsis thaliana*, this process unfolds along a clear developmental gradient. At the very tip of the root, new cells are born. As these cells move upward, they enter the elongation zone before finally reaching the differentiation zone, where they stop growing and differentiate into, for example, root hairs or water-transporting cells. A network of hormonal signals helps guide this progression. Peptide hormones, in particular, have emerged as key regulators of root zonation. For instance, ROOT GROWTH FACTOR1 sustains meristem activity by shaping PLETHORA transcription factor gradients and modulating reactive oxygen species dynamics (Yamada et al. 2020). Other sulfated peptides, such as PHYTOSULFOKINE and PLANT PEPTIDE CONTAINING SULFATED TYROSINE1 (PSY1), possess complementary roles in root development (Matsuzaki et al. 2010). While the phytohormones auxin and cytokinin define the transition from division to elongation, the mechanisms that determine the duration of the elongation phase are still poorly understood.

The clues needed to unravel this mechanism may lie in the interplay between signaling pathways and metabolism. Flavonols, a class of specialized metabolites, have been implicated in growth regulation and modulating auxin transport, suggesting that metabolic state influences development (Lewis et al. 2011; Daryanavard et al. 2023). PSY1 has emerged as a potential regulator of elongation dynamics, although its downstream effectors have remained elusive.

In a study published in this issue of *The Plant Cell*, **Ercoli and colleagues (Ercoli et al. 2026)** now identified PSY1 as a key regulator of root growth, controlling the extent of cell elongation prior to differentiation. Using the transcriptional reporter *ProPSY1:GFP*, they found that *PSY1* is expressed in the root differentiation zone but not in the epidermis. In contrast, its receptors, the *ROOT ELONGATION RECEPTOR KINASES* (*PSYR/REK*), with the exception of *PSYR1*, are broadly expressed across root tissues, including epidermal cell types. This spatial separation suggests that PSY1 may function as a non–cell-autonomous signal, coordinating growth by acting on cells still undergoing elongation.

The authors next found that PSY1 promotes root length: for example, the loss of PSY1 results in shorter primary roots due to premature exit from the elongation phase, leading to reduced mature cell size. In contrast, increased PSY1 levels, either through overexpression or exogenous treatment with a synthetic version of PSY1, prolong the elongation phase, allowing cells to reach larger final sizes. Detailed cellular analyses revealed no changes in meristem size or cell division rate, indicating that PSY1 does not promote cell proliferation. Instead, PSY1 specifically regulates the extent of cell elongation.

To investigate how PSY1 controls growth zonation, the authors performed RNA sequencing on roots treated with synthetic PSY1 and identified flavonol biosynthesis as a significantly enriched pathway. Upregulated genes included the flavonoid transporter *DTX35*, the RHAMNOSE SYNTHASE *RHM1/ROL1*, and the *3-KETOACYL-COA THIOLASE* isoform *KAT5*, suggesting that PSY1 triggers a rapid surge in the production and mobilization of flavonols.

Linking the production of flavonols to growth regulation, the authors finally demonstrated that PSY1 regulates root growth zonation through the modulation of ROS homeostasis and auxin signaling (Fig. 1). Flavonols perform a dual role in a cell: they scavenge reactive oxygen, which normally act as a “stop” signal for growth, and they dampen the growth inhibitory effects of auxin. By lifting these internal “brakes,” PSY1 creates a chemical environment that allows cells to maintain their expansion. Overall, the observed accumulation of flavonols has the potential to bridge the gap between peptide signaling and growth regulation.

**Figure 1:**
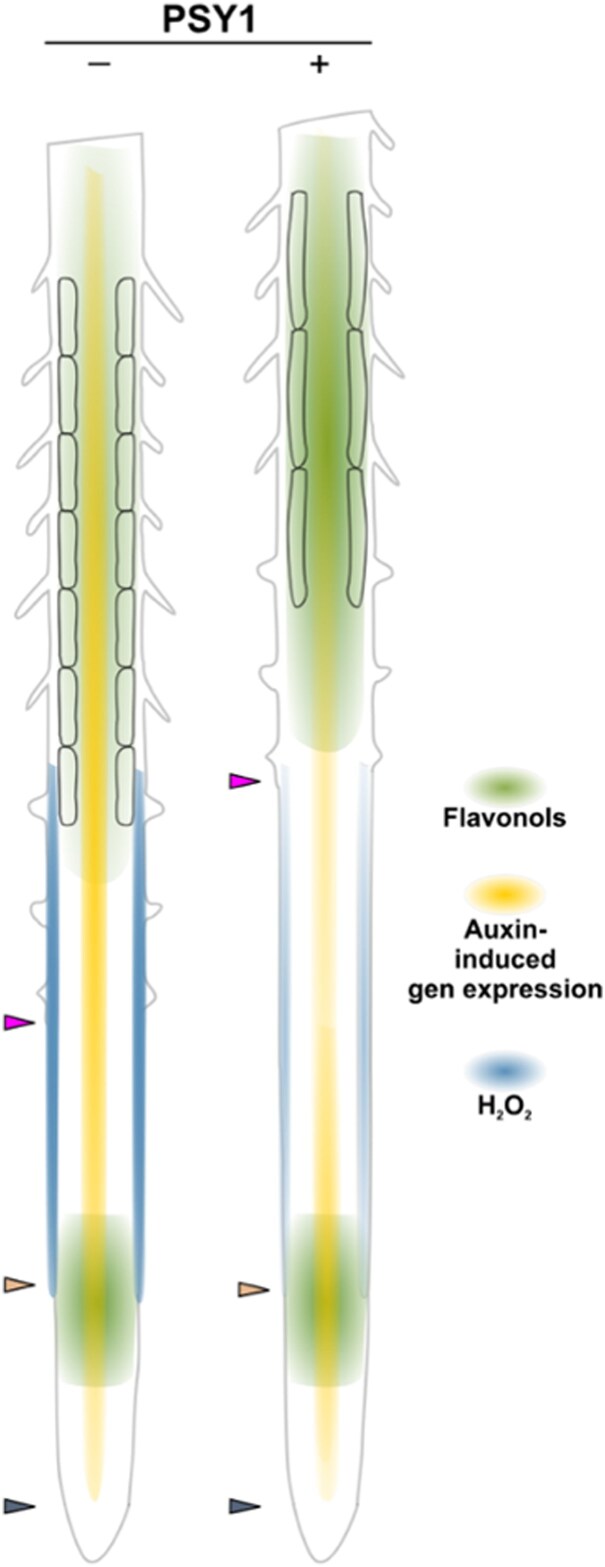
Schematic summary of PSY1 function in root growth. Elevated PSY1 levels promote root elongation by increasing mature cell size and delaying differentiation, as evidenced by root hair initiation occurring farther from the root tip. PSY1 also stimulates flavonol biosynthesis in the differentiation zone and the accumulation of flavonol glycosides in root exudates, which are required to reduce H_2_O_2_ levels in the epidermis of the elongation and differentiation zones. Additionally, auxin-responsive gene expression is decreased in the stele of the differentiation zone. Purple arrowheads indicate the quiescent center (QC), pale orange arrowheads the transition from meristem to elongation zone, and magenta arrowheads the end of the elongation zone, marked by root hair emergence (figure taken from Ercoli et al. 2026).

Together, these findings revealed a sophisticated regulatory circuit where a peptide signal from the differentiation zone activates specialized metabolism to manage developmental transitions. By modulating the internal balance of reactive oxygen species and auxin through flavonol biosynthesis, PSY1 determines the magnitude of cell elongation. This mechanism determines the topology of the root, defining where the differentiation zone ends and the elongation zone begins, ensuring the root reaches its full potential as it navigates the soil.

## Recent related articles in *The Plant Cell*

Zhou et al. 2024 investigated how ethylene regulates cell wall establishment during root growth in rice. They showed that ethylene, via ETHYLENE-INSENSITIVE3-LIKE1 (*OsEIL1*), activates *CELLULOSE SYNTHASE-LIKE C* (*OsCSLC*)- and *CELLULOSE SYNTHASE A* (*OsCESA*)-mediated polysaccharide biosynthesis, linking hormone signaling to cell wall remodeling that restricts cell elongation in coordination with auxin.
